# Druggable Drivers of Lung Cancer

**DOI:** 10.18632/oncotarget.1223

**Published:** 2013-08-14

**Authors:** Shameem Fawdar, Zoe C. Edwards, John Brognard

**Affiliations:** Signalling Networks in Cancer Group, Cancer Research UK Manchester Institute and The University of Manchester, Manchester, UK; Signalling Networks in Cancer Group, Cancer Research UK Manchester Institute and The University of Manchester, Manchester, UK; Signalling Networks in Cancer Group, Cancer Research UK Manchester Institute and The University of Manchester, Manchester, UK

Cancer genomic sequencing is ushering in an era of targeted and personalized therapeutic approaches for the treatment of cancer. Targeted therapies are yielding impressive response rates in patients, leading to longer progression-free survival and less severe side effects than those associated with traditional chemotherapeutic regimens. Treatment of EGFR mutation-positive lung cancer patients with EGFR inhibitors, or BRAF^V600E^-positive melanoma patients with a combination of RAF and MEK inhibitors, serve as impressive validators of this approach [1, 2]. However, the number of patients that can be treated with targeted therapies is limited by our understanding of the genetic drivers that are required to maintain cancer cell survival and proliferation [3]. To tackle this enormous challenge presented to the research community we applied a targeted genetic dependency approach to discover functionally relevant somatically mutated proteins required to maintain lung cancer cell survival and growth [4]. Utilizing cancer genomic sequencing data from the Sanger Institute we depleted six lung cancer cell lines of all somatically mutated proteins to discover novel genetic dependencies and identify low frequency driver mutations [5]. Given the impressive effort of The Cancer Genome Atlas (TCGA) and the International Cancer Genome Consortium (ICGC), among others, to catalogue all somatically altered genes in over 50 different cancers and over 500 cancer cell lines, our approach can be broadly applied to elucidate novel pharmacologically targetable mutationally activated enzymes. Alternatively, our strategy could be used as an approach to stratify patients for treatment with already existing therapies, such as MEK inhibitors. Our screen is broadly applicable and provides a potential clinical strategy to go from cancer genomic data, to genetic dependency identification, to administration of targeted therapies tailored to the patient's tumor. As n-of-1 clinical trials become a real possibility one could envisage isolating tumor cells from a biopsy, performing next-generation sequencing, establishing a cell line and screening all somatic mutants to identify individual Achilles' heels that can be targeted therapeutically. While cost prohibitive at this stage, the continual drop in prices of siRNA screening libraries, robotics, and next-generation sequencing means that bringing such screening efforts to the clinic could become a realistic prospect. In addition, the same strategy may be applied to circulating tumor cells, from which cell lines can be derived, presenting an opportunity to circumvent the more costly and invasive acquisition of cancer cells through biopsies. Targeted genetic dependency screens are an effective way to uncover low frequency oncogenes that can serve as targets for therapeutic intervention for tumors of any origin. The mutation frequency for the genes we identified ranged from 2-10% of lung cancers; given the frequency of lung cancer in the population, these targets could be exploited by pharmaceutical companies for drug development. Indeed PAK5 mutations have been identified in 9% of lung adenocarcinomas and if approximately half of these were gain-of-function mutations (we observed 4 out of 7 PAK5 mutants had increased activity compared to wild-type PAK5), this would represent a sizable patient population (approximately 65,000 patients/year globally) that could benefit from targeted inhibition of the PAK5 kinase [6]. Therefore the mutational activation frequency for PAK5 would mirror that observed in the EML4-ALK kinase rearrangement, where patients showed unprecedented response from treatment with the ALK inhibitor crizotinib [7]. An important finding from our study was the identification of mutations that act in concert to hyper-activate a specific pathway. For example, in one cancer cell line we observed an accumulation of activating mutations that converge towards the same central pathway to confer a proliferative advantage to the cell; specifically, we observed that the intermediately activating mutation BRAF^L597V^ can cooperate with PAK5^T538N^ and NRAS^Q61K^ to drive and maintain tumor growth via the RAF/MEK/ERK signaling axis. Thus a patient presenting to the clinic with a similar mutational profile would preferentially benefit from a pan-RAF or MEK inhibitor rather than a single BRAF-specific inhibitor. Furthermore, by identifying the individual hyper-activated components of the pathway, a combination therapy of targeted pharmacological inhibitors may prove to be more successful and better tolerated by the patient. In summary, targeted genetic dependency screens represent a valuable strategy to identify low frequency oncogenic mutations and can complement more traditional bioinformatic approaches; these tend to be selective for loss-of-function mutations as alterations in key residues required to maintain enzymatic activity are those most likely to be predicted as damaging [8]. As such platforms become increasingly used, we can foresee a future where a majority of cancer patients will be diagnosed on the basis of their tumor mutation profile and consequently receive the appropriately tailored treatment.

**Figure 1:**
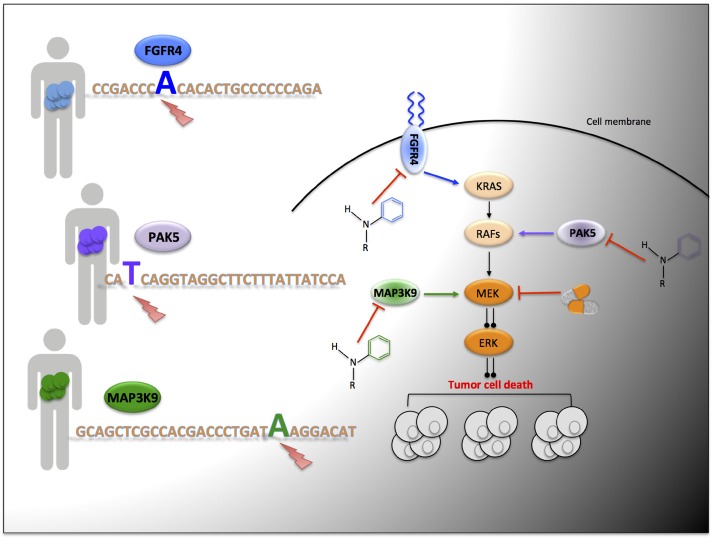
Targeted genetic dependency screen to identify novel actionable mutations; mutated FGFR4, MAP3K9 and PAK5 are illustrated examples Patient tumors are exome sequenced and treatment is stratified based upon the mutational profile of each individual. Treatment options include novel pharmacological compounds to specifically inhibit driver oncogenes, and/or targeting the main downstream hyper-activated pro-proliferative signaling pathway, for example inhibition of MEK.
